# Stent angioplasty of narrowed portocaval shunt in Budd Chiari syndrome: a case report

**DOI:** 10.1186/1757-1626-2-1

**Published:** 2009-01-01

**Authors:** Nilesh Doctor, Vidhyachandra Gandhi, Sharad Shah, Maharra Hussain, Shaji Marar, Sujith Philip

**Affiliations:** 1 Department of Gastrointestinal Surgery, Jaslok Hospital & Research Centre, Mumbai, India; 2 Department of Gastroenterology, Jaslok Hospital & Research Centre, Mumbai, India; 3 Department of Interventional Radiology, Jaslok Hospital & Research Centre, Mumbai, India

## Abstract

**Background:**

Hepatic vein thrombosis (Budd-Chiari Syndrome) is a rare disorder resulting from an obstruction to the outflow of blood from the liver. Early decompression is needed to prevent liver dysfunction and death. Radiological intervention includes angioplasty of stenosis and webs and the placement of transjugular intrahepatic portosystemic shunts (TIPPS). Side-to-side portacaval shunt (SSPCS) remains the gold standard for achieving good long-term results.

**Case presentation:**

A 37-year old lady underwent side-to-side portacaval shunt for Budd Chiari syndrome. She had early shunt blockage and this was successfully treated with the placement of a metallic stent across the shunt.

**Conclusion:**

At five years, she remains asymptomatic, with normal liver functions, no ascites, and normal flow through the stent on Colour Doppler examination.

## Background

Budd-Chiari syndrome is characterized by hepatic venous outflow obstruction, which often leads to death as a result of portal hypertension and liver failure. Patients with membranous occlusion of the major hepatic veins can be treated by percutaneous placement of a metallic stent via a transjugular or transhepatic approach [1]. In patients with a significant caval obstruction caused by a hypertrophied caudate lobe, a metallic vascular stent can be placed in the narrowed tract of the inferior vena cava, before shunt surgery, by means of a transfemoral or transjugular venous approach [1]. Side to side portacaval shunts have excellent long-term results and remain the procedure of choice in some units [2]. Stents can also be used to maintain the patency of surgical shunts that have been made to bypass the obstruction to the blood flow from the splanchnic circulation to the inferior vena cava via the liver. We report a long term follow up of stent angioplasty of a narrowed porta caval shunt in a patient with Budd Chiari Syndrome.

## Case presentation

A thirty seven year old female presented with a history of progressive distension of the abdomen with pedal edema and yellow discolouration of the eyes of two weeks duration. On examination she was found to be icteric with an enlarged, firm liver and gross ascites. Liver function tests revealed a conjugated hyperbilirubinemia with mildly deranged transaminases and alkaline phosphatase. Ultrasonography (USG) with Colour Doppler showed an enlarged liver with a hypertrophied caudate lobe, a 1.1 cm portal vein with hepatopetal flow, an occluded right hepatic vein with middle and left hepatic veins patent only in their proximal parts and a patent but narrowed inferior vena cava, compressed by the caudate lobe, together with free fluid in the abdomen. Splenic and superior mesenteric veins were normal. Liver biopsy confirmed the diagnosis of Budd Chiari syndrome. Prothrombotic workup did not reveal any obvious cause for the Budd Chiari syndrome.

**Figure 1:**
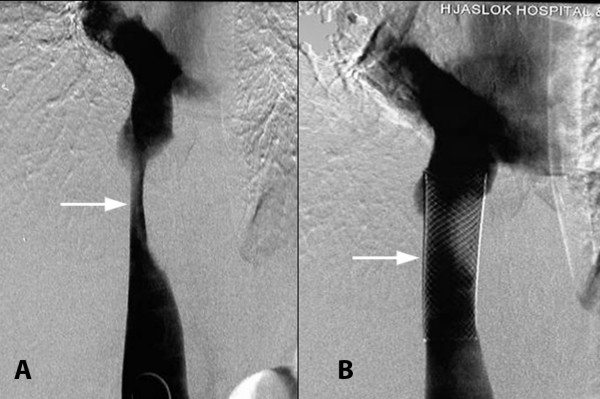
**A. Narrowed IVC with non visualization of hepatic veins**. B. IVC stent in situ.

Transjugular venogram showed 90% narrowing of the IVC with a 14 mmHg gradient across the narrowing and non-visualization of the hepatic veins beyond their origins. The hepatic veins could not be cannulated, suggestive of complete occlusion at the ostia. (Figure 1:A). An 18 × 63 mm WALL STENT was placed across the narrowed segment in the IVC followed by balloon dilatation using a 16 mm balloon (Figure 1:B).

**Figure 2:**
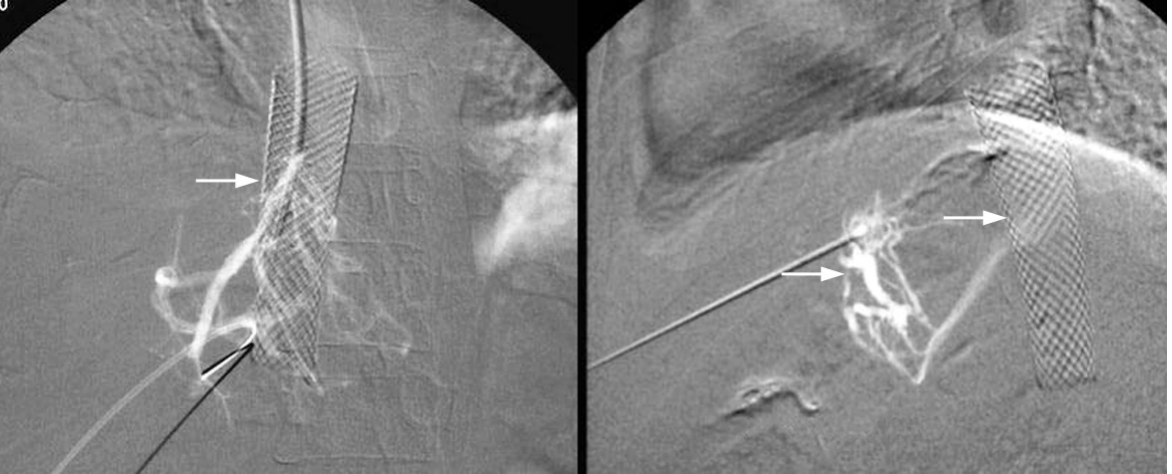
**Percutaneous transhepatic venogram revealed left hepatic vein replaced by multiple collaterals, IVC stent in situ**.

Percutaneous transhepatic venogram through the left hepatic vein under USG guidance showed the left hepatic vein was patent only in its proximal portion, the distal 2–3 cms being completely occluded and draining through multiple collaterals. IVC stent was in situ (Figure 2). The right hepatic vein was completely occluded. The middle hepatic vein also showed long-segment total occlusion (> 3 cm).

**Figure 3:**
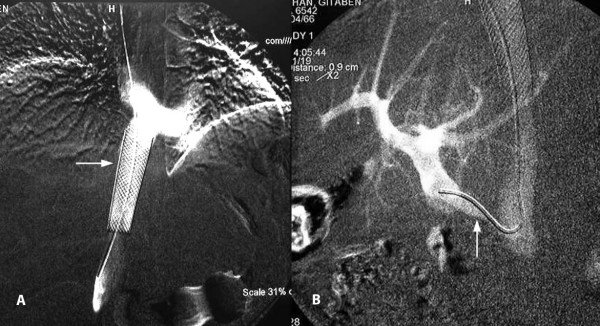
**Transjugular venogram A. Patent IVC Stent B. Narrowed portocaval graft**.

Recanalisation of the left hepatic vein was attempted, but failed owing to the long and fibrous nature of the occlusion. The patient then underwent a side-to-side portocaval shunt using the right external iliac vein as H-graft. Postoperative recovery was uneventful and she was anticoagulated during that time. Six weeks after surgery, she was re-admitted with pedal edema and ascites. Doppler evaluation showed patchy flow through the shunt. Venogram through a transjugular approach revealed patent IVC stent. There was narrowing of the portosystemic graft with a gradient of more than 15 mmHg near its IVC end (Figure 3:A, B).

**Figure 4:**
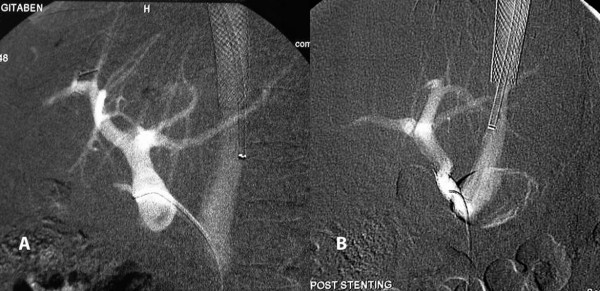
**Transfemoral venoplasty A. Pre dilatation of the graft**. B. Widened, patent graft post dilatation with stent in situ.

Considering the acute angulation of the portocaval shunt with the IVC, a transfemoral venous approach for venoplasty and stenting was thought to be more appropriate. Following predilatation of the graft with a 5 mm angioplasty balloon (Figure 4A), an 8 mm × 30 mm self-expanding nitinol stent was placed in the graft covering its portal and caval ends. Post dilatation was carried out by a 8 mm balloon. Post stenting portal venogram showed good flow through the graft into the IVC (Figure 4:B).

The portocaval gradient dropped to < 5 mmHg post stenting. Subsequently she was anticoagulated with warfarin. The patient has been on regular follow up since then and is asymptomatic for five years.

## Discussion

BCS is characterized by manifestations of portal hypertension, inferior vena cava occlusion or both [1]. It produces liver damage due to intense congestion, leading to development of cirrhosis. To prevent congestion, various nonsurgical and surgical procedures have been described.

A variety of surgical procedures can be used for decompression of the liver, including mesoatrial, mesocaval and portocaval shunts and liver transplantation. If there is no thrombosis in the inferior vena cava, a side-to-side portocaval shunt (SSPCS) is the procedure of choice in the early and middle stages of the disease [2]. Direct portocaval shunts have a thrombosis rate of 0.2%, compared with occlusion rates 24–53% for mesocaval shunts with synthetic grafts. However, autologous internal jugular vein grafts have patency rates comparable with those of portocaval shunts [3]. Mesoatrial shunts are preferred if there is a greater than 75% narrowing of the vena cava [4]. However, the patency rate of mesoatrial shunts is low, Slakey et al. [5] reporting a primary shunt patency rate of only 46%. To improve the shunt patency Orloff et al. [2] introduced a high-flow combination shunt, consisting of a side-to side portocaval shunt and a cavoatrial shunt. Results of this combined form were promising. Orthotopic liver transplantation is indicated in patients with chronic BCS who have cirrhosis and patients with a failed portosystemic shunt procedure. It has a 5-year survival rate of 69% [6]. Blockage of the shunts performed to decompress the liver can be a life-threatening event and is usually associated with excessive variceal hemorrhage. Recently percutaneous interventional techniques, including angioplasty, fibrinolysis, Self expanding metallic stents (SEMS) and TIPPS, have gained attention in the treatment of BCS and shunt occlusion [7]. Angioplasty has a high success rate in BCS due to congenital obstruction. For restenosis, balloon dilatation can be performed. As angioplasty simulates intimal hyperplasia, the number of balloon dilatations must be limited [7]. Xu et al. [8] advocated the use of metallic stents for such angioplasty failures. SEMS placement makes the dilatation more definitive.

The drop in venous pressure is more marked and it keeps the vessel patent for a longer time than angioplasty. But growth of a hyperplastic intima around the wire may reduce the diameter of the inferior vena cava. The stent may also cross the outlet of the hepatic vein, which results in an increase of turbulent flow and may lead to thrombosis [9]. TIPPS can be performed in BCS patients to improve the clinical condition while waiting for orthotopic liver transplantation. The TIPPS procedure is relatively safe and effective but its long-term patency is limited. Fortunately TIPPS stenosis responds well to reinterventions such as balloon dilatation and stents [10]. There are limited cases in the literature of percutaneous procedures secondary to a failed shunt, as most would become eligible for transplant. In our patient, we placed a SEMS in a narrowed portocaval shunt. As an interventional procedure, SEMS produced a good long term result with clinical improvement and patency. Therefore SEMS could be the procedure of choice in occluded portocaval shunts. We propose the use of SEMS as a salvage procedure for blocked side to side portocaval H-grafts before consideration of more radical procedures like liver transplantation.

## Abbreviations

TIPSS: Transjugular Intrahepatic Portosystemic Shunt; SSPCS: Side-to-side portacaval shunt; USG: Ultrasonography; BCS: Budd Chiari Syndrome; SEMS: Self expanding metallic stent

## Competing interests

The authors declare that they have no competing interests.

## Authors' contributions

Authors ND and MH – revising the article critically for important intellectual content. Authors VG and SP – drafting the manuscript. Author SS – medical management of the patient and revision of the article. Author SM – performed the angioplasty stenting and also helped in revising the article.

## Consent

Written informed consent was obtained from the patient for publication of this case report and accompanying images. A copy of the written consent is available for review by the Editor-in-Chief of this journal.
